# Location-Aware Wi-Fi Authentication Scheme Using Smart Contract

**DOI:** 10.3390/s20041062

**Published:** 2020-02-15

**Authors:** Yongle Chen, Xiaojian Wang, Yuli Yang, Hong Li

**Affiliations:** 1 College of Information and Computer, Taiyuan University of Technology, Taiyuan 030024, China, , yangyuliyyl@126.com (Y.Y.); 2 Institute of Information Engineering, Chinese Academy of Sciences, Beijing 100089, China

**Keywords:** authentication, blockchain, channel state information, smart contracts

## Abstract

Advanced wireless technology in Internet of Things (IoT) devices is increasing and facing various security threats. The authentication of IoT devices is the first line of defense for the wireless network. Especially in a Wi-Fi network, the existing authentication methods mainly use a password or digital certificate, these methods are inconvenient to manage due to certificate issuance or prone to be attacked because passwords are easily cracked. In this paper, we propose a location-aware authentication scheme using smart contracts to ensure that IoT devices can securely perform Wi-Fi network authentication. The scheme adopts the concept of secondary authentication and consists of two phases: the registration phase, which is mainly designed to complete the generation of the public and private keys, and to link the device information with its related device information; the authentication phase, which is mainly designed to determine whether the requesting device is within a legal location range. We use the smart contract to ensure the credibility and irreparability of the authentication process. Analysis of the attack model and the attacks at different stages proves that this certification scheme is assured, and the simulation results show that the overhead introduced by this scheme is acceptable, this scheme can provide greater security for the Wi-Fi authentication of IoT devices.

## 1. Introduction

With advanced 5G in Internet of Things (IoT) technology, a large number of embedded devices have data processing and network connectivity capabilities to provide smarter services. Gartner Inc. predicts that by 2020, the total number of IoT devices will reach 20.4 billion [1]. The boom in IoT devices and technologies has dramatically changed our daily lives in terms of efficiency and convenience.

However, the rapid growth of IoT devices is also facing many security threats [2,3]. IoT devices can be subject to a variety of malicious attacks, including traditional internet attacks and specially designed attacks against IoT devices. Unlike traditional computer networks, IoT systems are large-scale and can interact with physical environments, and security threats to IoT systems can lead to more serious hazards. For example, the Stuxnet worm against industrial IoT systems has caused significant damage to Iran’s nuclear facilities [4]. Mirai malware can efficiently scan IoT devices and infect vulnerable devices that use default factory settings or weak password encryption. After being infected by viruses, the device becomes a botnet robot and launches high-strength botnet attacks under the hacker’s commands. In addition, unauthorized access to IoT systems pose a serious threat to user privacy [5]. Especially in the smart home environment, the most pervasive network is the Wi-Fi network [6], and security issues such as privacy and confidentiality have always been the most vulnerable part of the wireless network [7]. Using a Wi-Fi cracker and other tools can easily crack passwords. Once a malicious device is accessed, it will pose a great threat to devices in the same LAN. To ensure that devices can join the network securely, this paper focuses on the network authentication of IoT devices in a WLAN environment.

There is no uniform security standard for IoT devices, Commercial Off-The-Shelf (COTS) IoT devices are protected by security schemes from various manufacturers. However, due to cost and complexity, these security solutions are insufficient to protect devices [8]. While traditional security solutions present several methods for handling a wide variety of security threats, they are not deployable and usable due to resource constraints and the need for human-computer interaction.

As a core component of the Bitcoin system [9], the blockchain maintains a distributed database that records all transaction data by using blocks of special data structures and prevents tampering with historical data. Blockchain attracts a great deal of attention from academics and practitioners, such as finance, healthcare, and government, because of its unique attributes, such as distribution, security, anonymity, privacy, and tamper resistance. Recently, blockchain technology has also been widely used in IoT systems. Slock.it [10] enables a smart electronic locker to be unlocked by using devices carrying appropriate tokens; in the energy field, the integration of the IoT and the blockchain ensures that machines can automatically buy and sell energy based on user-defined criteria [11].

The main way for the current IoT device to connect to Wi-Fi use the router’s SSID and password or digital certificate, the management is inconvenient and is vulnerable to attack. Considering the blockchain anti-tampering and the automatic execution of smart contracts, blockchain will be a better solution to IoT devices access authentication.

In this paper, we propose a location-aware authentication scheme using a smart contract to ensure that IoT devices can securely perform Wi-Fi network authentication. This method adopts the concept of secondary authentication, which not only requires a password, but also associates an IoT device authentication with other IoT devices, and uses the hard-to-forge physical layer channel state information (CSI) information which can be captured by a specific model of wireless card, such as a IWL5300 corresponding to the physical location as a judgment, using the smart contract to ensure the credibility and irreparability of the authentication process. This scheme is divided into two phases: The first phase is the registration phase, which is mainly to complete the public key and private key generation of the requesting device and to link the device information with its related device information. The second phase is the authentication phase. Challenge–response architecture is used to confirm that the requesting device correctly executes the commands sent by Access Point (AP), confirming the legality of the information returned by the requesting device and its related device, determining the similarity between the CSI information of the requesting device and the CSI information returned by the related device, thereby determining whether the requesting device is within a legal location range.

The main contributions of this paper are as follows:

- We designed an authentication scheme for IoT devices access to Wi-Fi network using CSI information combined with smart contracts.
- Challenge–response architecture is utilized in the authentication process to determine whether the device is in the legal range by using the location-related physical layer CSI information as an out-of-band feature. In this process, the aggregated signature is used to ensure the correctness of information transfer during the authentication process.
- We designed three smart contracts to ensure the confidentiality of the key issued during the registration phase, the security of requesting device and its related devices information, ensure the identity information query, public key query and aggregate signature verification are performed correctly during the authentication phase, and ensure that the IoT device will respond correctly when the storage space is insufficient.

The rest of this paper is organized as follows. In Section 2, we present the related works to IoT security and authentication. The application scenarios and general ideas of the authentication scheme are introduced in Section 3. In Section 4, we propose the authentication framework in detail and describe the techniques involved in the scheme. The security of the proposed authentication scheme is briefly analyzed in Section 5 and the performance of the scheme was evaluated by simulation experiments of Section 6. Finally, we summarize our work in Section 7.

## 2. Related Work

The security of devices in the IoT environment is becoming more and more prominent. Notra et al. proved that the security of IoT devices lacks basic security by invading multiple smart home devices [12]. Depending on the specific conditions of different IoT devices, all IoT devices may be subject to certain types of attacks [13].

Some work is trying to adapt the traditional internet authentication system to the internet of things environment. For example, Salman et.al proposed an identity-based IoT authentication scheme by introducing some concepts of software defined network (SDN) [14], the central SDN controller can monitor and manage all network elements and converts different technology-specific identities from different silos into shared identities and authenticates devices and gateways, but the cluster security is still an open issue because of the programmable and dynamic features of SDNs [15]. Pranata et al. proposed a framework for authentication, authorization, and access control for IoT environments based on tokens, Public Key Infrastructure (PKI), and encryption, which focus on using minimal computing resources, use de-centralized architectures and one party public key credentials, but this paper did not include the implementation of this method and its performance measurement [16]. Muhamed et al. proposed a new heterogeneous self-organizing wireless sensor network user authentication and key agreement scheme, the proposed scheme enables remote users to securely negotiate session keys with common sensor nodes using a lightweight key agreement protocol and ensures mutual authentication between users, sensor nodes and gateway nodes (GWN) [17]. Jan et al. proposed a lightweight mutual authentication scheme in the IoT environment, which is a payload-based encryption scheme that uses a four-way handshake mechanism to verify the identity of participating objects, using the constrained application protocol (CoAP) allows customers to view resources residing on the server in an energy-efficient manner, but they only analyzed the problems related to eavesdropping, DoS, replay, and resource exhaustion attacks, the problems with key distribution and limited sensor node resource still exist [18]. Jia et al. designed an identity-based anonymous authentication key agreement protocol for the mobile edge computing (MEC) environment, the protocol implements mutual authentication in a single message exchange round and ensures user anonymity and untraceability, but they did not consider other security properties affected by changes in environmental factors [19,20]. Chen et al. proposed a new three-party password-based authentication key exchange protocol to ensure the security of key exchange in wireless communication, their communication cost is acceptable but they used personal smart devices instead of arbitrary IoT devices for evaluation [21]. These methods basically use the idea of traditional authentication methods using cryptographic primitives for authentication in the IoT environment. These methods not only need to consider the issue of key distribution and management under traditional circumstances, but also need to consider whether these methods introduce new issues in the IoT environment.

In order to overcome the problem of key distribution and management based on cryptography, some researchers are studying the method of using the information on the perception layer for security authentication [22]. Fu et al. proposed an inter-domain communication authentication mechanism based on the internet of things, which uses pictures taken by IoT devices for authentication. Their mechanism achieved high usability and efficiency but it assumes that IoT devices that need to be authenticated must have a camera and can take pictures on their own [23]. Zhang et al. proposed a low-complexity framework for protecting IoT identity authentication and implementing secure communication, extracting small random differences between transceivers to create unique Radio Frequency (RF) fingerprints and determine user identity. The randomness of the wireless channel between the two users is used as the encryption key, but how to resist an attack is still an issue to be considered in Radio Frequency Fingerprinting (RFF) identification and key generation [24]. Amin et al. proposed an architecture suitable for distributed cloud environments and proposed an authentication protocol that allows registered users to securely access all private information from all private cloud servers. The protocol is superior than its counterparts with respect to various parameters but must rely on a smartcard [25]. Chen et al. proposed an improved protocol to solve the problem of authentication for vehicular ad hoc network (VANET) by using a smartcard [26]. Although these methods do not rely too much on cryptographic methods and remove the impact of complex and insecure factors such as key exchange, they require additional hardware to get the necessary information, such as cameras, smart cards, etc., which are not supported by IoT devices that cannot use this hardware.

In order to solve the problem of the centralization of authentication, the blockchain attracts great attention from academics and practitioners because of its unique attributes such as distribution, security, anonymity, privacy, and tamper resistance. Liang et al. proposed a micro-blockchain based geographical dynamic intrusion detection for vehicle-to-everything and their approach is more accurate than traditional Intrusion Detection System (IDS) [27]. Sanda et al. proposed a method of Wi-Fi access authentication using blockchain technology, they show that blockchain can be used as logs of users’ statistics and provides a platform for regional applications [28]. Lin et al. proposed a peer-to-peer (P2P) knowledge market to make knowledge tradable in edge-AI enabled IoT, blockchain was used for secure and efficient knowledge management and trading for the market [29,30]. Zhu et al. proposed a blockchain-based IoT identity framework (BIFIT) for user-centered IoT smart home, blockchain was used to maintain the owner’s identity, owner’s private key sign, and the signature of the identity of the devices. [31]. In order not to use the credential service provider (CSP) architecture that must be involved in the federated identity management process, Mell et al. design a Federated Identity Management (FIM) system that enables the direct user to RP authentication and attribute transfer without the involvement of a third party by using the smart contract [32]. Lin et al. proposed a blockchain-based secure mutual authentication system, because blockchain provides privacy and security guarantees, and the use of smart contracts makes the framework scale well [33]. Jiang et al. proposed a new blockchain-based WLAN mesh network security access scheme authentication protocol, which solves the problem of requiring key delivery and requiring a central server in IEEE 802.11i and IEEE 802.11s authentication processes. The method treats the user’s authentication request as a transaction and treats all the authentication records in the mesh network as a public ledger to effectively monitor malicious attacks [34]. The above references show that blockchain technology and smart contracts can be applied to the IoT environment, and can increase security without introducing a huge overhead, so it is feasible to apply it to the Wi-Fi network authentication of IoT devices.

Our approach applies the idea of blockchain technology between devices and devices, supplemented by hard-to-forge physical layer CSI information related to the physical location, adopting the concept of secondary authentication. This decentralizes the identity authentication mechanism of IoT devices to other IoT devices by using the smart contract of the blockchain to ensure the credibility and irreparable modification of the authentication process and the legitimacy of the devices in the authenticated process, thus making the authentication process of the IoT device more secure and reliable.

## 3. Preliminaries

### 3.1. Application Scenario

**Figure 1:**
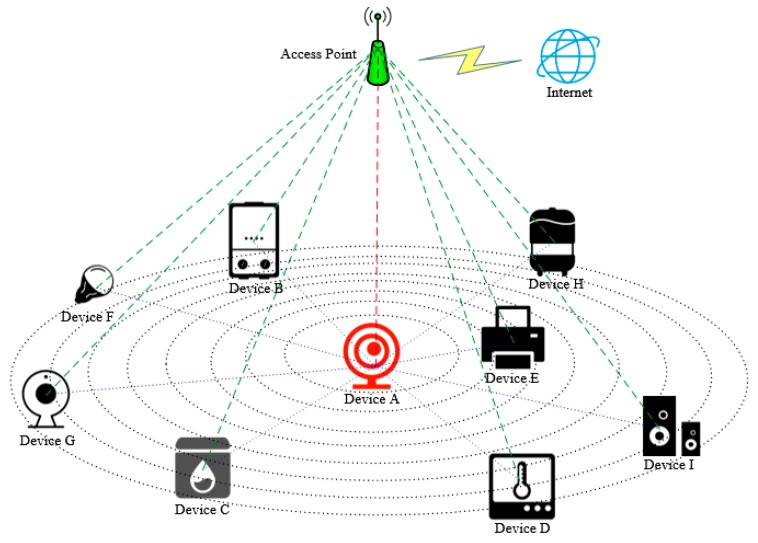
Application scenario of the proposed authentication architecture.

In this paper, we mainly consider the network access authentication of IoT devices in smart home scenarios. In this scenario, assume that all devices that need to access the network have wireless networking functions and Bluetooth functions. Devices that need to perform network authentication must enter the password to generate a public and private key pair, record the device information, and the information of the related devices that is close to it utilizing smart contract to successfully register, and then initiate the network authentication request. The authentication phase uses the challenge–response system to confirm that the requesting device correctly executes the random instruction, and uses the CSI information returned by the related devices to confirm whether the requesting device is in a legal location. The aggregate signature guarantees the correctness of the returned CSI information. As shown in Figure 1, Device A is a newly added device that needs to perform network authentication. All devices B-I have already accessed the network through identity authentication. Device A and other devices have Bluetooth function and can sense the Bluetooth RSSI of the surrounding devices. Our goal is to propose a method for judging the legitimacy of IoT devices that need to be connected to the network, preventing illegal devices from appearing in the protected WLAN and attempting to access the network to ensure the security of the entire network.

The main idea of the proposed authentication scheme is to adopt the idea of secondary authentication, which not only requires a password, but also associates the authentication of an IoT device with other IoT devices, and uses the physical location. The hard-to-forge physical layer CSI information is used as a credential to judge whether the requesting device is legal. The credibility and irreparable modification of the authentication process are ensured by the use of smart contracts in the blockchain. See Section 4 for details.

### 3.2. Threat Model

Assume that an attacker attempts to introduce malicious device X into a smart home environment, and attempts to pretend to be legitimate device Y for Wi-Fi network authentication, and device X can initiate a network authentication request to the AP. We only consider the case where the illegal device X is different from the physical location of the legitimate device Y, the physical location refers to where the device is in space at a time, different physical locations indicate that the two devices are not physically in the same location. If the physical locations of two devices are close enough, they must be in the same space, and because the CSI information we use is fine-grained, if a device wants to impersonate another device, it must be in the same position as a legitimate device. Two devices in the same position are equivalent to stacking together. In the current smart home environment, two stacked devices are easily found by the administrator due to their size not being small enough.

Traditional internet attacks are also applicable to IoT devices, and because of the limited computing and storage resources of IoT devices, attacks on IoT devices may be easier. The attacks that may be encountered during the authentication process are as follows.

DoS attack: The illegal device continuously sends authentication requests during the registration and authentication phases; exhausting network bandwidth or computing resources lead to the legal device not being able to access the network.

Message replay attack: The illegal device intercepts the message and resends the message. Replay attacks may occur during the process of returning device addresses and public keys in the registration phase and during the process of responding to random instruction in the authentication phase.

Impersonation attack: An illegal device steals the identity of a legitimate device and uses it to eavesdrop on this WLAN environment.

Man-in-the-middle attack: The rogue device places itself between the requesting device and the AP, forwarding request and response messages, tampering with and eavesdropping on the authentication process.

Password guessing attack: During the registration phase, the attacker obtained the public and private keys using a password guessing method.

Location-related attack: Illegal device attempts to access this WLAN within an illegal location.

## 4. Authentication Scheme

### 4.1. Overview

**Figure 2:**
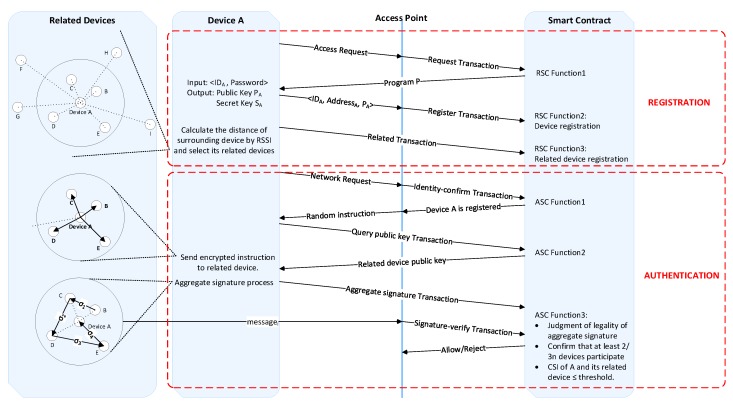
Overview of our proposed authentication framework. Abbreviations: ASC, authentication smart contract; RSC, register smart contract; CSI, channel state information.

The authentication scheme proposed by us is mainly aiming at the Wi-Fi network authentication process in the current smart home environment, which mainly adopts the authentication methods such as password authentication or digital certificate, they are simple and the management is inconvenient, but the security is not high at the same time. We propose a location-aware Wi-Fi network authentication scheme using smart contract. This method adopts the idea of secondary authentication and is divided into two phases: The first phase is the registration phase, which is mainly to complete the public key and private key generation and to link the device information with its related device information. The second phase is the authentication phase, the challenge–response architecture is used in this phase, confirming the legality of the information returned by the requesting device and its related device, determining the similarity between the CSI information of the requesting device and the CSI information returned by the related device, thereby determining whether the requesting device is within a legal location range. We use the smart contract to constrain the behavior of the device and ensure the correct execution of the whole process of identity authentication policy. The overview of our proposed authentication framework is shown in Figure 2.

a. Registration phase.

We adopt the idea of secondary authentication. The first stage is the registration phase, a valid password is required to register successfully. The registration phase is mainly to complete the public key and private key generation and to link the device information with its related devices information. The requesting device A that requests access for the first time needs a password to generate the address of this device on the blockchain and generates the device’s own private key, followed by the process of recording device information and related device information. This process is constrained by a smart contract called register smart contract (RSC). Take device A for Wi-Fi network authentication as an example, the specific steps of this process are as follows:

(1) Device A initiates a request <Name_A_, ID_A_> try to access AP. Name_A_ is the name of device A and ID_A_ is the ID of device A.
(2) After receiving this request, the AP initiates a Request Transaction and the smart contract RSC monitors this request information, executes Function 1: Sends a program P which can be run by the requesting device to device A.
(3) A receives the program P and enters <ID_A_|password> into this program P.
(4) After the program P confirms that the password inputted is correct, a pair of unique public key P_A_ and a private key S_A_ are generated for the device A. The private key of the device is a random number randomly generated by the device A and keep by itself. The public key of the device is calculated by the ECC algorithm. The hash of P_A_ is used as the address Add_A_ of the device A, then <Add_A_|P_A_> is sent to the AP.
(5) After receiving the information, the AP initiates a register transaction, which triggers the Function 2 of the smart contract RSC: Record the device information <Name_A_, ID_A_, Add_A_, P_A_>.
(6) Device A perceives the RSSI value of the Bluetooth of the surrounding devices and calculates the distance corresponding to the different devices, and selects the n devices closest to it as their related devices.
(7) Device A initiates a related transaction, which triggers the Function 3 of the smart contract RSC: Confirm whether the device in the related transaction has legal identity in the network environment (the related device is the device that was previously authenticated), and if the related device is confirmed as a legal device, record the related device information <Num., Distance, Related_dev_Address, Establishment_time, Failure_time>. Num. represents the number of the related device, Distance represents the distance from the related device to the requesting device, Related_dev_Address represents the address of the related device, Establishment_time represents the time when the requesting device establishes an association relationship with the related device, and Failure_time represents the failure of the association time.

b. Authentication phase

The second phase of this scheme is the authentication phase, which uses location information for authentication. The challenge–response system is used to confirm that the requesting device correctly executes the random command sent by the AP, and uses the aggregate signature to confirm the legitimacy of the information returned by the requesting device and its related device. In order to determine whether the requesting device is within the legal location range, it determines whether the CSI information of the requesting device is similar to the CSI information returned by its related devices. If it is within the legal scope, it approves the device to access the network. Otherwise, it refuses this device to access the network. This process is constrained by a smart contract called authentication smart contract (ASC). The specific steps of this process are as follows:

(1) Device A requests to access the network.
(2) After receiving the request information, the AP initiates an identity-confirm transaction, triggering Function 1 of the smart contract ASC: confirming that device A is registered legally and querying the related devices information of device A.
(3) The AP sends a random instruction (RI) (TargetID, Speed, Duration) encrypted by the device A public key to device A, and the content of the random instruction is to collect m packets at a rate of n packets per second, devices around device A need to follow this command to return their respective CSI values.
(4) Device A receives this random instruction and initiates a query public key transaction which triggers Function 2 of smart contract ASC: Query the public key of its related device, encrypt the random instruction with the corresponding public key of its related device and send it out.
(5) Device A’s related devices receive this instruction, decrypts it with their own private key to get the original instruction, collects m packets at the rate of n packets per second (message) according to the instruction. Signs the message with its own private key and encrypts the signed message using the AP’s public key and sends it to the AP. The related devices perform aggregation signature according to the sequence number, and then the last related device sends this signature to device A, device A collects its CSI information according to the instruction and then performs final signature process. Device A initiates an aggregate signature transaction and puts the final signature on the chain.
(6) The AP initiates a signature-verify transaction, which triggers Function 3 of the smart contract ASC: Confirm whether the aggregate signature in the transaction is legal and whether at least 2/3 of n devices participate in the aggregate signature, whether the similarity of CSI collected by device A and its related devices are within the threshold (which is described in detail in Section 4.4.3), and message matching, where n is the total number of related devices of device A. If all the conditions are met, the AP allows device A to access the network. Otherwise, device A is denied access to the network.

Among them, all transactions are sent by the corresponding initiator with their own private key signature. In the following subsection, we detail the most important design modules involved in the authentication process: related devices selection, aggregate signature, CSI processing, block structure, and smart contract.

### 4.2. Related Devices Selection

In the process of selecting related devices, the RSSI of the Bluetooth is used to estimate the distance of the device around the requesting device, and the devices closest to the requesting device are selected as its related devices. From the version of Bluetooth 2.1, a Bluetooth HCI interface called Inquiry_With_RSSI is introduced and leads to a way to measure RSSI without being directly connected to the device. By using this command, all surrounding Bluetooth devices can be measured at once and a list of sensed Bluetooth devices and their RSSI values can be produced [35].

After obtaining the RSSI of the surrounding devices, taking into account the effects of multipath effects in the indoor environment, we adopt the channel fading model of the indoor environment mentioned in [36]:

RSSI(dbm) = −(10Nlog_10_d − P) (1)

(2) $$\mathrm{distance}\left[m\right]=\;10^{\frac{RSSI-P}{-10N}}$$

where N is the signal fading constant, which reflects the characteristics of RSSI, and attenuated according to the environment, d/distance is the distance from the signal source, P is the signal strength when the transmitter and the receiver are separated by 1 m, and RSSI is the received signal strength.

Using this channel fading model, we can get the distance corresponding to the RSSI of the surrounding related devices. After obtaining the distance according to the RSSI calculation, we calculate the standard deviation of the distance over a period of time, and select the device with a small standard deviation and a short distance as the related device. The device blocked by people or obstacles can cause the distance calculation to become longer and fluctuated because the RSSI intensity will decrease and fluctuate. This device will not be selected as the relevant device, and other devices with short distance calculations and small standard deviations will be selected as the related device.

### 4.3. Aggregate Signature

In this paper, we used the method of aggregate signature for device identification and personal data proof in [37], and the correctness of this method has been proved. The specific steps for the aggregation signature for this paper are as follows:

(1) Initialization of the public parameters params and master secret key msk (Setup(*λ*) → (params,msk)). Given the security parameter *λ*, let *G*, *G_T_* be two cyclic multiplicative groups of prime order *p*, the generator of *G* is *g*, given a blinear map *e*: *G* × *G* → *G_T_*, randomly select *α*, *β* ∈ *_R_Z_p_* and compute *h*_1_ = *g^α^*, *h*_2_ = *g^β^*. Let *H*_1_,*H*_2_,*H*_3_:{0,1}* → *G*, *H_4_*:{0,1}* → *Z_p_* be four cyptographic hash functions. Set the public parameters as params = (*p*,*G*,*G_T_*,*e*,*g*,*h*_1_,*h*_2_,*H*_1_,*H*_2_,*H*_3_,*H*_4_) and the master secret key as msk = (*α*,*β*).
(2) Generate the private key for network *i* *sk_i_* (KeyGen_1_ (params, msk, $I_{i}^{\left(N\right)}$) → *sk_i_*), the Setup and KeyGen_1_ process is performed by the smart contract RSC. Compute *g_i_* = *H*_1_($I_{i}^{\left(N\right)}$), $C_{i}^{\left(1\right)}$ = $g_{i}^{\alpha }$, $C_{i}^{\left(2\right)}=\beta$, the private key with $I_{i}^{\left(N\right)}$ is *sk_i_* = ($C_{i}^{\left(1\right)},C_{i}^{\left(2\right)}$), $I_{i}^{\left(N\right)}$ is the identity of network *i*. Since the aggregation signature process is performed only under one LAN in this paper, we set *i* = 1.
(3) Generate private key for related devices *sk*_1,*j*_ (KeyGen_2_(params, *sk*_1_, $I_{j}^{\left(D\right)}$) → *sk*_1,*j*_), Compute *g*_*j*,0_ = *H*_2_($I_{j}^{\left(D\right)},0$), *g*_*j*,1_ = *H*_2_($I_{j}^{\left(D\right)},1$), $D_{1,j}^{\left(1\right)}=C_{i}^{\left(1\right)}\cdot g_{j,0}^{C_{1}^{\left(2\right)}}$, $D_{1,j}^{\left(2\right)}=C_{1}^{\left(1\right)}\cdot g_{j,1}^{C_{1}^{\left(2\right)}}$, Set the private key with $I_{1}^{\left(N\right)}$ as *sk*_1,*j*_ = ($D_{1,j}^{\left(1\right)},\;D_{1,j}^{\left(2\right)}$), where *j* represents the the number of related devices, *j* = 1,2,3,..,n. The generation logic of the device’s key is implemented in program P, which executes on the device to generate a public and private key pair for this device.
(4) Each device aggregates in turn to generate the final aggregate signature $\sigma_{1,n}$, the generate process follows Sign_j_(params, *sk*_1,*j*_, $\sigma_{i,j-1}$, *m_j_*) → $\sigma_{1,j}$. Where *m_j_* is the CSI information collected by the related device *j*, *j* ∈ [1,*n*]. For *j* = 1, $I_{1}^{\left(D\right)}$ choose a string w that has not been used by other signatures. For *j* > 1, $I_{j}^{\left(D\right)}$ check string w has not been used by other signatures, then the device randomly chooses *t_j_* ∈ *_R_Z_p_*, Compute *g_w_* = *H*_3_(*w*), *a_j_* = *H*_4_(*m_j_*, $I_{j}^{\left(D\right)},w$.), $B_{1,j}^{\left(1\right)}=g_{w}^{tj}\cdot D_{1,j}^{\left(1\right)}\cdot {\left(D_{1,j}^{\left(2\right)}\right)}^{aj}$, $B_{1,j}^{\left(2\right)}=$ *g^tj^*. For *j* = 1, $\sigma_{1,0}=⊥$, $\sigma_{1,1}=(B_{1,1}^{\left(1\right)}\cdot B_{1,1}^{\left(2\right)},w)$. For *j* > 1:
  (3) $$\sigma_{1,j-1}=\;(S_{1,j-1}^{\left(1\right)},S_{1,j-1}^{\left(2\right)},w)$$
  The generation of $S_{1,j}^{\left(1\right)}$ and $S_{1,j}^{\left(2\right)}$ is shown in Equations (4) and (5):
  (4) $$S_{1,j}^{\left(1\right)}=S_{1,j-1}^{\left(1\right)}\cdot B_{1,j}^{\left(1\right)}=\prod_{j^{\prime }=1}^{j}B_{1,j\prime }^{\left(1\right)}=g_{w}^{\sum_{j^{\prime }=1}^{j}t_{j\prime }}\cdot g_{1}^{j\alpha }\cdot \prod_{j^{\prime }=1}^{j}g_{j\prime }^{\beta }\cdot g_{1}^{\alpha \sum_{j^{\prime }=1}^{j}a_{j\prime }}\cdot \prod_{j^{\prime }=1}^{j}g_{j\prime }^{\beta a_{j\prime }}$$
  (5) $$S_{1,j}^{\left(2\right)}=S_{1,j-1}^{\left(2\right)}\cdot B_{1,j}^{\left(2\right)}=\prod_{j^{\prime }=1}^{j}B_{1,j\prime }^{\left(2\right)}=g^{\sum_{j^{\prime }=1}^{j}t_{j\prime }}$$
(5) The validity of the final aggregate signature is verified to determine whether $\sigma_{1,n}$, has been tampered with, and whether the CSI data transmitted by each related device is the original data (Verif(params, $I_{1}^{\left(N\right)}$, $\left\{I_{j}^{\left(D\right)}\right\}$_*j*∈[1,*n*]_, $\sigma_{i,n}$, {*m_j_*}_*j*∈[1,*n*]_) → {“ACCEPT”,”REJECT”}). Verify that whether the following formula is true, if it true, the aggregate signature is considered legal, otherwise, it is considered illegal. Verif process is done by the smart contract ASC.
  (6) $$e\left(S_{1,j}^{\left(1\right)},g\right)=e\left(H_{3},S_{1,j}^{\left(2\right)}\right)\cdot e\left(H_{3}\left(I_{1}^{\left(N\right)}\right),h_{1}^{n}\cdot h_{1}^{\overset{n}{\underset{j=1}{\sum }}a_{j}}\right)\cdot e\left(\overset{n}{\underset{j^{\prime }=1}{\prod }}H_{2}\left(I_{j}^{\left(D\right)},0\right)\cdot H_{2}{\left(I_{j}^{\left(D\right)},1\right)}^{a_{j}},h_{2}\right)$$

**Figure 3:**
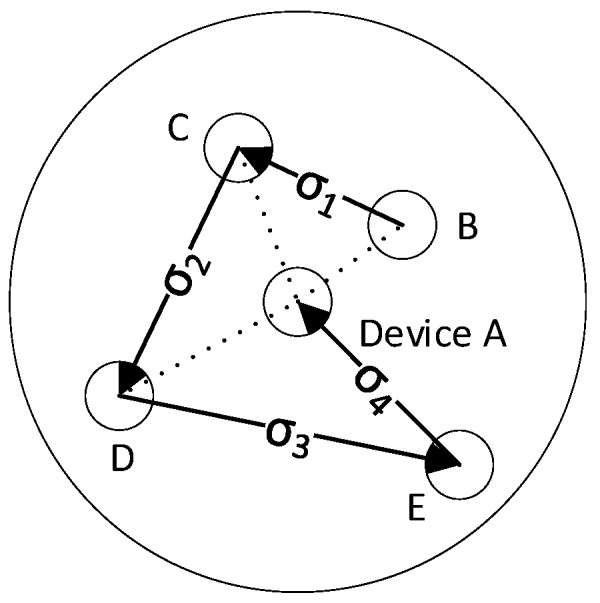
The process of aggregating signature.

In this paper, the order of the aggregated signatures is based on the sequence number. The sequence number is obtained by sorting the distances calculated by the RSSI from small to large. The closer the distance is, the smaller the sequence number is. If a device has not responded after 2 s in the aggregation signature process, skip this device and continue the signature process of the next device. The aggregation signature process is shown in Figure 3.

In the Verify phase, the smart contract ASC first verifies that the final aggregate signature is legal, and secondly determines whether at least 2/3 of devices participate in the aggregate signature.

### 4.4. CSI Process

#### 4.4.1. Channel state information (CSI)

OFDM technology is widely used in current wireless communication, and OFDM utilizes multiple carrier frequency technology and parallel transmission technology, which can extend the time of symbol transmission, thereby enabling the signal to be improved in multi-path transmission resistance. Channel information at the subcarrier level becomes available. More specifically, CSI, as physical layer information, describes in more detail how the signal propagates from the transmitter to the receiver than RSS, thus more accurately describing the effects of channel gain, scatter, fading, and power attenuation. CSI is restricted to be obtained with a specific model of network card, so not all models of network cards support obtaining CSI data. A specific model of network card used in conjunction with the custom modified firmware and open source Linux wireless drivers can be used to obtain CSI data. In this paper, we collected the CSI by using an open-source tool named CSI Tool [38] which is built on the Intel Wi-Fi Wireless Link 5300 802.11n MIMO radios; we collected the CSI data in 2.4 GHz frequency with 20MHz bandwidth. A set of CSI information is a set of discrete sampling values of channel frequency response within the specified bandwidth that set the interval of the frequency sampling to a certain value. In a narrowband flat fading channel, a frequency domain OFDM system can be modeled as follows:

*Y* = *HX* + *N* (7)

where *Y* and *X* are the received vector and the transmit vector, respectively, and *H* and *N* are the channel matrix and the additive white Gaussian noise (AWGN) vector, respectively. The CSI data structure is an m × n × 30 matrix. m is the number of transmit antennas, and n is the number of receive antennas. Using the 2.4 GHz frequency and 20 MHz bandwidth, the CSI information of 30 subcarriers, which were acquired using the Intel 5300 NIC, can be expressed as follows:

(8) $$H(k)=||H(k)||e^{j∠H(k)}$$

where *H*(*k*) represents the channel matix of the k-th subcarrier, and ||*H*(*k*)|| and ∠*H*(*k*) represent the amplitude and phase of the first subcarrier, respectively. The 30 subcarriers can be expressed as *H* = [*H*_1_,*H*_2_,*H*_3_,….*H_i_*,…*H_n_*]^*T*^, *i* ∈ [1,30].

#### 4.4.2 Content of Message

In order to more accurately represent the physical state of the wireless signal of the IoT device, we use the data of 30 subcarriers between the transmitter antenna and a receiver antenna. The number of packets varies according to the random instruction. In order to reduce the packet load, we do not directly transfer the original CSI data, but convert the CSI data into a grayscale image in which size can be reduced to 8 bytes, which is greatly reduced compared to the original CSI data stream. Each data packet takes the data of the 30 subcarriers of the first transmitting antenna and the first receiving antenna. The number of data packets P varies according to the random instruction received by the device. Therefore, for each group of returned CSI data, a 30 × P matrix *M*_30×*P*_ which contains data of 30 subcarriers of each packet in P packets can be formed. The structure of the matrix *M*_30×*P*_ is shown in formula (9), where *d*_*m*, *n*_ represents the data of the n-th subcarrier of the m-th data packet.

(9) $$M_{30\times P}=\left(\begin{array}{cccc} d_{1,1} & d_{2,1} & \cdots & d_{P,1}\\ d_{1,2} & d_{2,2} & \cdots & d_{P,2}\\ \vdots & \vdots & \ddots & \vdots \\ d_{1,30} & d_{2,30} & \cdots & d_{P,30} \end{array}\right)$$

This matrix *M*_30×P_ can be converted into a grayscale image, that is, the matrix *M*_30×*P*_ is normalized into an image matrix by using min–max normalization, the value of each element in the normalized matrix is in the range of 0 to 1 (including 0 and 1), where 0 represents black and 1 represents white.

#### 4.4.3 Message Matching

Message matching occurs in Function 3 of the smart contract ASC to verify whether the requesting device is in the range around its related devices, and is located in step (6) in the authentication phase. Due to the nature of its open medium, wireless positioning is vulnerable to malicious attacks [39], considering heterogeneity in the hardware of the devices [40], we did not choose to use CSI directly to estimate the location of the device, instead, the CSI clustering results of multiple related devices are used to determine whether the device is within the legal location range.

The purpose of message matching is to determine the degree of similarity between the CSI of the requesting device and its surrounding related devices. If the degree of similarity is high, the device is considered to be legally located around its related device. Otherwise, the device may be considered to be out of the legal location and may be impersonated. Message matching mainly includes two aspects: packet number matching and CSI matching.

Packets number matching: The requesting device and its related devices collect CSI information according to the random instruction (TargetID, Speed, Duration). The random instruction RI has three parameters, and the TargetID is the request_ID of the request (request_ID, request_Domain) sent by the requesting device. The speed specifies that the CSI information is collected at a certain speed, such as 100 packets/second. The duration parameter specifies the duration of collecting CSI, such as 10 s. The purpose of the random instruction is to inform the participating devices to send their CSI information with some random parameters. The received CSI information is used as the message content. Considering the instability of the network and wireless transmission, we consider the gaps in the 50 packages to be acceptable fluctuations.

CSI matching: The CSI information of the requesting device is compared with the CSI information of its related device. First, use the PCA algorithm to reduce the dimension of all collected data, let the processed matrix of requesting device as *M*′ and the processed matrix of its related devices as *M_i_*. To determine the similarity between the matrix M’ and *M_i_*, we measure the similarity of the matrix using the correlation distance.

The matrix correlation coefficient is calculated as follows:

(10) $$\rho M_{i}M^{\prime }=\frac{\mathrm{cov}(M_{i},M^{\prime })}{\sqrt{D(M_{i})}\sqrt{D(M^{\prime })}}=\frac{E((M_{i}-EM_{i})(M^{\prime }-EM^{\prime }))}{\sqrt{D(M_{i})}\sqrt{D(M^{\prime })}}$$

The correlation distance is computed as follows:

(11) $$DM_{i}M^{\prime }=1-\rho M_{i}M^{\prime }$$

We take the correlation distance of 1 m as ‘*Thres*’. This threshold range determines the legal position range, when the correlation distance between matrix *M*′ and *M_i_* is less than *d*·*Thres*, we think that the relative position of this device and its related device *i* is legal. The formula for the calculation is as follows:

*D*_*M_i_M*′_ ≤ *d* · *Thres* (12)

The distance *d* is calculated based on the channel fading model of the RSSI information.

### 4.5. Block Structure

**Figure 4:**
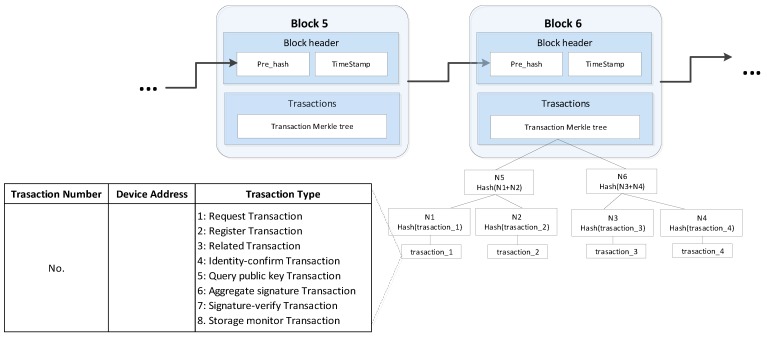
Block Structure.

The block structure used by this authentication scheme is shown in Figure 4.

The block structure consists of two parts: the block header and the transaction. The block header is composed of the hash value Pre_hash of the previous block and the TimeStamp. The transaction part is the root of the transaction Merkle tree. We store the transaction information in the form of a Merkle tree so that other devices can judge whether a transaction exists without downloading the entire block. In this paper, the operation of packaging the transaction into the blockchain is done by the AP.

There are 7 transaction types, namely request transaction, register transaction, vertical transaction, identity-confirm transaction, query public key transaction, aggregate signature transaction, signature-verify transaction, storage monitor transaction. Their corresponding numbers are identified in Figure 4.

### 4.6. Smart Contract

A smart contract is a set of programs that are automatically executed on the blockchain. Using the smart contract function of the blockchain, we can automate the execution of some pre-defined event-triggered programs deployed on the blockchain to control the IoT devices and ensure the legitimacy of the authentication process. The “smart contract” was proposed by Szabo in a pioneering paper in 1994 [41]. In order to ensure the credibility and tamper-resistant of the authentication process, three smart contracts are used in the proposed authentication scheme, smart contract for the registration phase(RSC), for the authentication phase(ASC), and for IoT storage capacity limitations(SSC).

*(1)* *Register smart contract, (RSC)*

As shown in Algorithm 1, the RSC has three functions. Function 1 is responsible for sending a program P that the device can run to the requesting device, thereby helping the device to generate the public key and private key pair. Function 2 is responsible for recording device information <Name_A_, ID_A_, Add_A_, P_A_>. Function 3 is responsible for confirming whether the device in the related transaction has a legal identity in the network environment (the related device is the device that was previously authenticated). If the related device is confirmed as a legitimate device, the related device information <num., distance, Related_dev Address, establishment time, failure time> is recorded.
**Algorithm 1** Register Smart Contract1: **function 1**2: (*Trigger Transaction: Request Transaction*3: *Input: Request ID and Name of the access device< Name_A_, ID_A_ >)*4:  Send a program P to ID_A_;5: **end function 1**6: **function 2**7: (*Trigger Transaction: Register Transaction*8: *Input: Basic information of the device)*9: Record device information<Name_A_, ID_A_, Add_A_, P_A_>;10: **end function 2**11: **function 3**12: (*Trigger Transaction: Related Transaction*13: *IInput: Information of the related device)*14: Record related device information<num., distance,15: Related_dev Address, establishment time, failure time>;16: **function 3**

*(2)* *Authentication Smart Contract, (ASC)*

As shown in Algorithm 2, the ASC has three functions. Function 1 is responsible for confirming that the requesting device is registered on the blockchain and querying the related information of the requesting device. Function 2 is responsible for querying the public key of its related devices, and encrypts the random information with this public key and sends it out. Function 3 is responsible for confirming whether the aggregated signature in this transaction is legal, whether there are at least 2/3 of n devices participating in the aggregate signature process, and whether the CSI similarity of device A and its related devices is less than the threshold we described in part D. If all the conditions are met, the AP allows requesting device to enter the network. Otherwise, the requesting device is denied access to the network.
**Algorithm 2** Authentication Smart Contract1: **function 1**2: (*Trigger Transaction: Identity-confirm Transaction**3: Input: Request address of the network access device Add_A_**4: Output: True or False)*5: **if** device was registered **then**6:  Query the number of related devices x;7:  **return** true;8: **else**9:  **return** false;10: **end if**11:**end function 1**12:**function 2**13: (*Trigger Transaction: Query public key Transaction*14: *Input: Request address of the network access device Add_A_*15:*Output:* Address of the related devices and the *corresponding public key)*16: Query public key about related devices;17: **return** <Add_i_, P_i_>18: **end function 2**19: **function 3**20: (*Trigger Transaction: Signature-verify Transaction*21: *Input: Aggregate signatures*
$\sigma_{A}$, *CSI of request device CSI_A_ and its related devices CSI_i_*22: *Output: True or False)*23: **if**
$e\left(S_{1,j}^{\left(1\right)},g\right)==e\left(H_{3},S_{1,j}^{\left(2\right)}\right)\cdot e\left(H_{3}\left(I_{1}^{\left(N\right)}\right),h_{1}^{n}\cdot h_{1}^{\sum_{j=1}^{n}a_{j}}\right)\cdot e\left(\prod_{j^{\prime }=1}^{n}H_{2}\left(I_{j}^{\left(D\right)},0\right)\cdot H_{2}{\left(I_{j}^{\left(D\right)},1\right)}^{a_{j}},h_{2}\right)$24:  **then**25:  **if** Number(participating in the aggregate signature) ≥$\frac{2}{3}$n **then**26:   **if** D_MiM’_ ≤ d · Thres **then**27:    **return** true;28:   **else**29:    **return** false;30:   **end if**31:  **else**32:   **return** false;33:  **end if**34: **else**35:  **return** false;36: **end if**37: **end function 3**

*(3)* *Storage Smart Contract, (SSC)*

Due to the limited storage capacity of IoT devices, as shown in Algorithm 3, when the storage capacity of a node reaches the limit, the storage capacity monitoring smart contract is triggered, and all other information except the necessary information is integrated into a log to be uploaded to the cloud backup or local external device backup and then delete the other information on that node.
**Algorithm 3** Storage Smart Contract1: **function 1**2: (*Trigger Transaction: Storage monitor Transaction*3: *Input: Transaction originator address Add_A_)*4: **if** Add_A_ initiates a Storage monitor Transaction request **then**5:  Back up data to the cloud or local, retain only the necessary data;6: **end if**7:**end function 1**

## 5. Security Analysis

In this section, we discuss the attacks that this authentication scheme may encounter. Any security design needs to address three major security requirements: confidentiality, integrity, and availability (CIA). In which, the three are interdependent and form an indivisible whole, any damage will affect the security of the whole system. Confidentiality prevents unauthorized users from accessing data, integrity prevents unauthorized modification of data, and availability ensures that authorized users can access data in a timely and accurate manner.

Confidentiality: The information exchange in the Wi-Fi network authentication process is encrypted by the public key generated during the registration phase, and can only be interpreted by the receiver with the specific private key.

Integrity: The challenge–response system guarantees that the data for each response is different. The aggregated signature guarantees the correctness of the returned information and ensures that the returned location information will not be tampered with.

Availability: The behavior of a device is constrained by smart contracts so that the identity authentication process can be performed according to the established policy.

Further, we discuss the security of this method from two aspects, one is to consider the stage and behavior of malicious attacks, and the other is to consider the security under different attack models.

### 5.1. Security Consideration

Assuming that the device that the malicious request accesses from is behaving abnormally, we consider attacks that may occur in five different phases:

Case 1: Program P was intercepted during the registration phase.

Case 2: The Wi-Fi password was compromised.

Case 3: Random instructions are intercepted during the authentication phase.

Case 4: The public key is intercepted when the public key is queried during the authentication phase.

Case 5: The message returned during the authentication phase aggregation signature process is intercepted.

For case 1, if program P is intercepted midway, the illegal device may pretend to be a legitimate device, but a Wi-Fi password is required to register.

For case 2, after the Wi-Fi password is inputed into the program P, the illegal device obtains a public key and a private key, but in the subsequent authentication phase, since the illegal device is not within the legal location range, there is no legal CSI information. Thus, it is impossible to authenticate successfully.

For case 3, the random instruction is encrypted with the public key of the requesting device. Only requesting device can unlock the content of the random instruction, the purpose is to confirm the identity of the requesting device and make sure it correctly executes this instruction. The random instructions are different each time, and the last intercepted information cannot be used for playback.

For case 4, the query public key is only for encrypting when sending information to the related device, and the interception of the public key itself is not threatening.

For case 5, the message returned by the aggregated signature is encrypted using the public key of the AP, and only the AP can know the true message information.

The reason for using the smart contract to complete the key generation function rather than the requesting device directly sends the Wi-Fi password to the AP and the AP performs the private key generation work because the private key may become easily intercepted and be used for camouflage in the process of issuing the private key by the AP. However, if a smart contract is used to deliver a private key, only the device can know its own private key, and the private key does not need to be transmitted over the network, so the security is greatly improved.

The reason why the aggregated signature is used in the authentication process is that the aggregated signature can ensure that the returned messages of all devices are legal, and it can be used to aggregate multiple signatures into one signature, effectively reducing the computational cost and communication overhead in the authentication process and speeding up the verification.

The three types of smart contracts used in the registration phase and the authentication phase are triggered by different types of transactions, which ensure the confidentiality of the key issued during the registration phase, the security of the recording requesting device and its related devices’ information. They ensure the information query in the authentication phase, the correct execution of the public key query and the aggregate signature verification, and ensure that the IoT device responds correctly when the storage space is insufficient.

### 5.2. For Thread Model

We briefly analyze the security of the proposed authentication scheme based on the types of possible attacks described in Section 3.2 of Section 3.

In a DoS attack, smart contracts control the whole process of authentication. They can analyze the traffic source and traffic size during the authentication process, and detect abnormal traffic in time to stop the DoS attack.

In a message replay attack, the replay attack that occurs during the registration phase can only get the device address and public key information, which are almost unhelpful for the attacker, but it is impossible to get the private key information. It is random during the authentication phase. It is also meaningless to intercept the information captured during the random instruction response process during the authentication phase, because the random instruction is changed each time, and the returned response information is encrypted so that the content cannot be arbitrarily falsified. The instructions will not be identical each time, and the replay attack will not produce the required data.

In an impersonation attack, the illegal device may have the public key and address of a device, but it cannot get the private key generated by the device itself, so the aggregate signature cannot be correctly performed, and it must not be correct. It will not respond correctly to the related device request initiated by other devices in the network, and the hard-to-forge physical layer CSI information related to physical location returned by this device must exceed the legal threshold range, so it is very easy to be discovered and eliminated. Since the authentication process is constrained by smart contracts, and the smart contract must be triggered by a specific transaction type, the unique key information of the device is used in the information interaction process to ensure that the requesting device is the same device from beginning to end.

In a man-in-the-middle attack, it can be detected using the RTT time, and the interactive information sent during when the authentication process is encrypted with the public key of the device itself or the AP. It cannot tamper with the information even if it is intercepted.

In a password guessing attack, during the registration phase, the function of the password is to obtain the public key and private key information of the device through the input program P. Firstly, the program P has measures taken when the password is input incorrectly, so as to ensure that the wrong password cannot frequently input, otherwise the malicious device will be alarmed. Secondly, even if the password information is obtained, since the device is not in the legal location range, the information of the associated device of the device cannot be obtained, and the registration phase cannot be completed, so that the operation of the identity authentication phase cannot be performed.

In a location-related attack, an illegal device attempts to access this WLAN within an illegal location. Firstly, if the device is not in the legal location range, the device cannot complete the registration process correctly, because the registration process requires the device to obtain RSSI information of other devices and register other devices as their related devices; secondly, even if the registration phase is successfully completed in the registration phase, in the authentication phase, device A needs to integrate the CSI information received by itself according to the random command into the aggregated signature. If this devices is not in a legal physical location, it will not generate legal CSI data, and thus the authentication process cannot be completed correctly.

## 6. Performance Evaluation

In order to provide better security, this Wi-Fi network authentication scheme brings computing and communication overhead on the smart home device. To evaluate this overhead, we simulated the solution in a smart home environment in stages. We used the Cooja [42] tool to simulate the smart home environment, the CSI collected in the real world is used as the input of the simulation environment, and the corresponding data are fed back at different stages of authentication. In addition, we also compared the packet overhead with the performance part of a (Block Chain)BC lightweight framework proposed in [43]. Although it is not specifically used for authentication, it is also a method of using blockchain in the smart home environment.

### 6.1. Packet Overhead

We have counted data packets’ lengths that transmitted at different stages, and compared them with the traditional method without using the scheme and the BC-based method in [42], the details are shown in Table I. The lengths of several different control frames used in the normal unencrypted Wi-Fi access authentication process according to the IEEE 802.11 protocol are as follows (the length of the optional part and the length of the variable part are not calculated): the beacon frame sent by the AP is 40 bytes, probe request frame is 28 bytes, probe response frame is 89 bytes, authentication frame is 34 bytes, association request frame is 32 bytes, and reassociation request frame is 38bytes.

Table 1 describes the packet payload using this scheme. Compared to the traditional method, the use of ciphertext transmission and multiple interactions increases the transmission load. Compared with the method in [43], our method significantly increases the packet load during the process of sending the program P from the AP to the device, it is the result of ensuring the security of the private key issued during the authentication process. However, this larger packet load needs to be sent only when the authentication is in its first initialization, after that, the program will be stored in the device and will not need to be sent again in the subsequent authentication process. Compared to the reliable security provided by this scheme, the packet load introduced by this scheme is acceptable.

### 6.2. Time Overhead

Limited edge computation and storage resources of IoT devices may cause calculation time to be relatively long with high latency [44].

**Figure 5:**
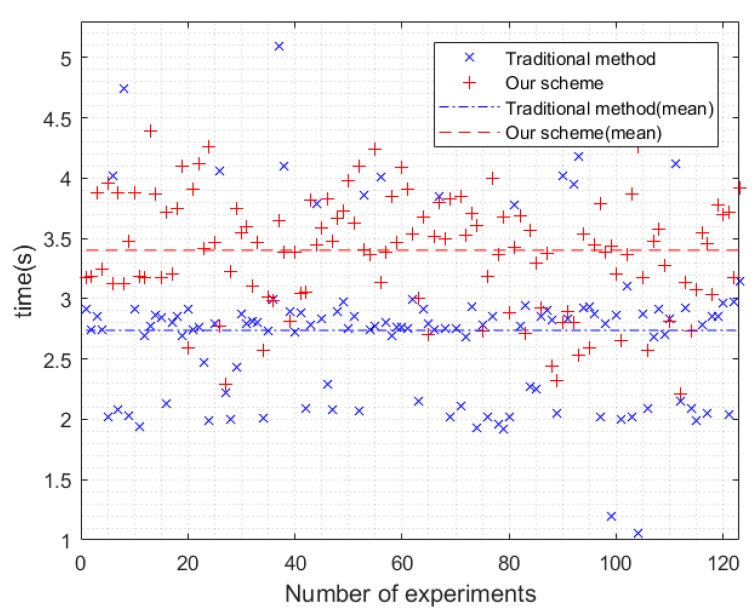
Time overhead compared to traditional method.

Time overhead compared to our scheme with a traditional method is shown in Figure 5. In Figure 5, the abscissa represents the number of experiments and the ordinate represents time. The red “+” represents the data of our scheme, the blue “×” represents the data of the traditional method, the red dotted line represents the mean of our scheme, and the blue dotted line represents the mean of the traditional method. We conducted a total of 123 experiments. We tallied the time delay of this method, synthesize delays caused by all transactions plus packet interaction during authentication, found related devices, CSI data collection, aggregate signature generation, data processing, and other overhead delays, from receiving incoming authentication requests to allow/deny device access is about 3.4028 s for 5 related devices, where the delay is more than 4 s mostly because of the more delay than usual due to the delay of other devices in the search for related device stage and the aggregate signature stage. We measured the time it took for IoT devices to access Wi-Fi using WPA2 PSK encryption in the current smart home environment as the traditional method, which has an average latency of 2.7375 s, as shown in Figure 5, the average latency of traditional method scheme is acceptable compared to 2.7375 s.

In summary, the overhead introduced by the smart contract-based Wi-Fi access authentication method is acceptable compared to the security it provided.

### 6.3. Authentication Performance

We listed the performance of this authentication method with different numbers of related devices (from 2 to 7), as shown in Table 2. For each specific number of related devices case, the total number of experiments is 500, including the not attack experiment for device access to Wi-Fi network at a legal location and the attack experiment for device access to Wi-Fi network at an illegal location. 

In order to accurately estimate what may happen when devices with different relative positions initiate Wi-Fi network access requests, we take each related device as the center and divide its surrounding area into 40 concentric circles, each concentric circle being 0.5 m apart, then randomly select the positions of the related devices according to the number of related devices. For different numbers of related devices, we switched the selected related devices group 5 times to ensure that the selected related device is different each time. After selecting the position of the related device, the CSI data corresponding to the related device is extracted from the database, and the legal device should be in the legal range under the current situation according to the selected related device position, so as to obtain the positions where the illegal devices may be. A random function is used to randomly select the positions of the intersections of the concentric circles as the positions where the requesting device may initiate the request, we assumed that the device at the selected position initiates a Wi-Fi network access request.

In Table 2, true positive (TP) means that the attack device is predicted as an attack device. True negative (TN) means that the not attack device is predicted as not attack device. False positive (FP) means that the not Attack device is predicted as an attack device. False negative (FN) means that the attack device is predicted as a not attack device. True positive rate (TPR) = TP/(TP + TN), accuracy = (TP + TN)/(TP + FP + FN + TN), precision = TP/(TP + FP), recall = TP/(TP + FN), F-1 score = (2 × P × R)/(P + R).

It can be seen from Table 2 that the precision of this method is maintained at 100%, this means that our method determines that the attack must be an attack, and there will not be a situation that the legal device is judged as an illegal device, which will reduce the availability of the IoT device. 

In the case of only two related devices, because the number of related devices is relatively small, the number of related devices that need to verify CSI during the message matching phase in the authentication process is easier to meet the conditions and is considered a legitimate device. Therefore, the attack that should have been judged as an attack has not been judged, resulting in a recall of only 91.84%. As the number of associated devices increases, the number of related devices that need to be verified during the message matching phase increases, thereby increasing the recall.

The accuracy increases with the increase in the number of related devices, but at the same time as the number of related devices increases, the time overhead also increases. When the number of related devices is 2, the accuracy is only 96%. When the number of related devices is 5, the accuracy is more than 99% and the time overhead is controlled below 4 s. This situation is acceptable under the conditions of the current number of devices in the smart home environment. Therefore, the number of related devices in this method should be at least 3, when N = 5, this method achieves a balance between accuracy and time overhead.

## 7. Conclusions

The Wi-Fi network authentication process in the current smart home environment mainly adopts password authentication or digital certificate authentication methods, the management is inconvenient and the security is not high. This paper proposes a location-aware Wi-Fi network authentication scheme using smart contract. The method adopts the idea of secondary authentication and is divided into two phases: the first phase is the registration phase, which is mainly to complete the public key and private key generation and to link the device information with its related device information. The second phase is the authentication phase, the challenge–response architecture is used in this phase, confirming the legality of the information returned by the requested device and its related device, determining the similarity between the CSI information of the requested device and the CSI information returned by the related device, thereby determining whether the requested device is within a legal location range. The security of this authentication scheme is analyzed by the proposed attack model and the attacks at different stages. The simulation results show that the overhead introduced by our proposed authentication scheme is acceptable relative to the security provided by the scheme.

## Figures and Tables

**Table 1:** Evaluation of package overhead. Evaluation of package overhead.

Package Flow	Our Scheme (Bytes)		Traditional (Bytes)	Package Flow	BC-Based (Bytes) [42]
From device to AP	Access Request	35	94	From devices to the miner	16
	<ID_A_, Add_A_, P_A_>	30			
	Network Request	35			
	message_i_	67			
From AP to device	Program P	5k	129	From the miner to the cloud	36
	Random instruction	38			
From device to device	Send encrypted instruction to related device	38	-	From the cloud to the miner	16
	Aggregate signature process	56			

**Table 2:** Performance of authentication. Performance of authentication.

Related Devices Number	Not Attack	Attack	TP	TN	FP	FN	TPR	Accuracy	Precision	Recall	F-1 Score	Time Overhead
2	255	245	225	255	0	20	0.4688	0.96	1	0.9184	0.9574	3.0089s
3	287	213	207	287	0	6	0.419	0.988	1	0.9718	0.9857	3.0933s
4	236	264	257	236	0	7	0.5213	0.986	1	0.9735	0.9866	3.1716s
5	237	263	259	237	0	4	0.5222	0.992	1	0.9848	0.9923	3.4028s
6	242	258	257	242	0	1	0.515	0.998	1	0.9961	0.9981	4.0010s
7	235	265	265	235	0	0	0.53	1	1	1	1	4.3297s
